# KCHO-1, a Novel Antineuroinflammatory Agent, Inhibits Lipopolysaccharide-Induced Neuroinflammatory Responses through Nrf2-Mediated Heme Oxygenase-1 Expression in Mouse BV2 Microglia Cells

**DOI:** 10.1155/2014/357154

**Published:** 2014-12-11

**Authors:** Dong-Sung Lee, Wonmin Ko, Chi-Su Yoon, Dong-Cheol Kim, Jinju Yun, Jun-Kyung Lee, Ki-Young Jun, Ilhong Son, Dong-Woung Kim, Bong-Keun Song, Seulah Choi, Jun-Hyeog Jang, Hyuncheol Oh, Sungchul Kim, Youn-Chul Kim

**Affiliations:** ^1^Inha Research Institute for Medical Sciences, Department of Biochemistry, Inha University School of Medicine, Incheon 400-712, Republic of Korea; ^2^Institute of Pharmaceutical Research and Development, College of Pharmacy, Wonkwang University, Iksan 570-749, Republic of Korea; ^3^Hanpoong Pharm & Foods Co., Ltd., Jeonju, 561-841, Republic of Korea; ^4^Department of Neurology, Inam Neuroscience Research Center, Sanbon Medical Center, College of Medicine, Wonkwang University, Iksan 570-749, Republic of Korea; ^5^Department of Internal Medicine, Wonkwang University College of Korean Medicine, Iksan 570-749, Republic of Korea; ^6^Department of Oriental Internal Medicine, Wonkwang Gwangju Oriental Medical Hospital, 543-8 Juwol Dong, Nam-gu, Gwangju 503-310, Republic of Korea; ^7^Institute for Cell Engineering, The Johns Hopkins University School of Medicine, Baltimore, MD 21205, USA; ^8^ALS/MND Center of Wonkwang University Korean Medical Hospital, 543-8 Juwol Dong, Nam-gu, Gwangju 503-310, Republic of Korea

## Abstract

The brain is vulnerable to oxidative stress and inflammation that can occur as a result of aging or neurodegenerative diseases. Our work has sought to identify natural products that regulate heme oxygenase (HO)-1 and to determine their mechanism of action in neurodegenerative diseases. KCHO-1 is a novel herbal therapeutic containing 30% ethanol (EtOH) extracts from nine plants. In this study, we investigated the antineuroinflammatory effects of KCHO-1 in lipopolysaccharide- (LPS-) treated mouse BV2 microglia. KCHO-1 inhibited the protein expression of inducible nitric oxide synthase (iNOS), iNOS-derived nitric oxide (NO), cyclooxygenase- (COX-) 2, and COX-2-derived prostaglandin E2 (PGE_2_) in LPS-stimulated BV2 microglia. It also reduced tumor necrosis factor-*α* (TNF-*α*), interleukin-1*β* (IL-1*β*), and IL-6 production. This effect was correlated with the suppression of inhibitor of nuclear factor kappa B-*α* (I*κ*B-*α*) phosphorylation and degradation and nuclear factor kappa B (NF-*κ*B) translocation and DNA binding. Additionally, KCHO-1 upregulated HO-1 expression by promoting nuclear translocation of nuclear factor E2-related factor 2 (Nrf2) in mouse BV2 microglia. Tin protoporphyrin (SnPP), an HO activity inhibitor, was used to verify the inhibitory effects of KCHO-1 on proinflammatory mediators and proteins associated with HO-1 expression. Our data suggest that KCHO-1 has therapeutic potential in neurodegenerative diseases caused by neuroinflammation.

## 1. Introduction

Neurodegeneration is the progressive loss of neuronal structure and function, leading to cell death. Neuroinflammation, or inflammation of the central nervous system (CNS), is a pivotal process that occurs in response to irritants, such as pathogenic infection, disease, and injury [1]. Further, it is a central mechanism regulating neurodegeneration in diseases. In recent years, the incidence of neurodegenerative disease has increased in aged populations, leading to increased research and the development of novel drugs to treat diseases such as Alzheimer's disease (AD), stroke, Parkinson's disease (PD), and amyotrophic lateral sclerosis (ALS) [1]. Microglia are myeloid-lineage cells residing in the nervous system and play a role in CNS immune response [2]. They are activated in response to stimuli or toxins, including lipopolysaccharide (LPS) and *β*-amyloid (A*β*) [3]. However, excessive activation of microglia leads to the secretion of a variety of proinflammatory cytokines and mediators, such as nitric oxide (NO), reactive oxygen species (ROS), inducible nitric oxide synthase (iNOS), cyclooxygenase-2 (COX-2), tumor necrosis factor-*α* (TNF-*α*), interleukin (IL)-1*β*, and IL-6 [4]. In addition, the transcription factor nuclear factor-*κ*B (NF-*κ*B) is activated in active microglia, thereby regulating the expression of many genes mediated and involved in immune and inflammatory response [5] and aggravating inflammatory conditions.

Recent studies have described the protective effect of heme oxygenase (HO)-1, through its ability to reduce oxidative stress in neuronal cells and consequently neuronal disease [6–9]. HO-1 is an inducible isoform of HO, which degrades heme to carbon monoxide, ferrous iron, and biliverdin/bilirubin [10, 11]. HO-1 and its byproducts are considered a potential target for the treatment of inflammatory diseases [12]. The expression of HO-1 is regulated by the transcription factor nuclear factor E2-related factor 2 (Nrf2). Under normal conditions, Nrf2 resides in the cytoplasm, where it binds Kelch-like ECH-associated protein 1 (Keap1). Upon stimulation, such as oxidative stress, Nrf2 is released from Keap1 and translocated into the nucleus, where it binds the antioxidant response element (ARE) on the HO-1 promoter [13]. When cells are subjected to inflammatory stimuli, they typically respond by inducing coordinated expression of phase II detoxifying enzymes, which activate numerous transcription factors, including Nrf2 [14]. Previous reports suggest that phytochemicals can regulate Nrf2 translocation by directly binding to Keap1, leading to the induction of cytoprotective and anti-inflammatory proteins, including HO-1 [15].

KCHO-1 is a novel herbal combination containing 30% ethanol (EtOH) extracts obtained from nine herbs, including* Curcuma longa*,* Salvia miltiorrhiza*,* Gastrodia elata*,* Chaenomeles sinensis*,* Polygala tenuifolia*,* Paeonia japonica*,* Glycyrrhiza uralensis*,* Atractylodes japonica*, and processed* Aconitum carmichaeli*. These herbs have been used in traditional medicine for many years [16–31]. In this study, we have investigated the antineuroinflammatory effects of KCHO-1 in LPS-stimulated BV2 microglia. We show that HO-1 expression plays a crucial role in mediating the antineuroinflammatory effects of KCHO-1.

## 2. Materials and Methods

### 2.1. Chemicals and Reagents

Dulbecco's modified Eagle's medium (DMEM), fetal bovine serum (FBS), and other tissue culture reagents were purchased from Gibco BRL Co. (Grand Island, NY, USA). Tin protoporphyrin IX (SnPP IX; HO inhibitor) was obtained from Porphyrin Products (Logan, UT, USA). Cobalt protoporphyrin IX (CoPP induces HO-1 expression), Trolox, and all other chemicals were obtained from Sigma Chemical Co. (St. Louis, MO, USA). Primary antibodies, including HO-1, COX-2, iNOS, p65, p50, p-I*κ*B*α*, I*κ*B*α*, and Nrf2, and secondary antibodies were purchased from Santa Cruz Biotechnology (Heidelberg, Germany). Enzyme-linked immunosorbent assay (ELISA) kits for PGE_2_, TNF-*α*, IL-6, and IL-1*β* were purchased from R&D Systems, Inc. (Minneapolis, MN, USA).

### 2.2. Materials of Extracts

The* C. longa*,* C. sinensis*,* P. tenuifolia*,* P. japonica*,* G. uralensis*, and* A. japonica* were purchased from the Wonwkang herb Co. (42, Hongsamhanbang-ro, Jinan-eup, Jinan-gun, Jeollabuk-do, Korea) in August 2013. The* S. miltiorrhiza* and* G. elata* were purchased from DONGKYUNG PHARM. Co., Ltd. (331-13, Maehwaguin-ro, Boeun-eup, Boeun-gun, Chungcheongbuk-do, Korea). A processed* A. carmichaeli* was purchased from the Hanpoong Pharm & Foods Co., Ltd. (333-24, Palbok-dong 1-ga, Deokjin-gu, Jeonju-si, Jeollabuk-do, Korea). All voucher specimens ID were deposited at the Hanpoong Pharm & Foods Co., Ltd. (*C. longa* (HP2013-10-01),* S. miltiorrhiza* (HP2013-10-02),* G. elata* (HP2013-10-03),* C. sinensis* (HP2013-10-04),* P. tenuifolia* (HP2013-10-05),* P. japonica* (HP2013-10-06),* G. uralensis* (HP2013-10-07),* A. japonica* (HP2013-10-08), and processed* A. carmichaeli* (HP2013-10-09)). The* C. longa* (4 kg),* S. miltiorrhiza* (4 kg),* G. elata* (4 kg),* C. sinensis* (2 kg),* P. tenuifolia* (2 kg),* P. japonica* (2 kg),* G. uralensis* (2 kg),* A. japonica* (2 kg), and processed* A. carmichaeli* (1 kg) were mixed, pulverized, and extracted in 30% ethanol (KP) for 3 h at 84~90°C, concentrated using a rotary evaporator and lyophilized. The entire procedure was repeated two times. KIOM-4 was dissolved in dimethyl sulfoxide (DMSO), the final concentration of which did not exceed 0.1%.

### 2.3. HPLC Analysis

The KCHO-1 samples were analyzed by reverse phase-high performance liquid chromatography (HPLC) using a Sykam HPLC from Sykam Gmbh. (Eresing, Germany) equipped with a S7131 reagent organizer, S2100 solvent delivery system, S7511 vacuum degasser, S5200 sample injector, and S3210 UV/Vis detector. HPLC-grade acetonitrile was purchased from Burdick & Jackson (Honeywell, Muskegon, MI, USA). Data processing was carried out using ChromStar DAD (GPC) software from Sykam Gmbh. (Eresing, Germany). An inertsil-ODS3 column from GL Science Inc. (Torrance, CA, USA) was used as the stationary phase (150 mm × 4.6 mm; particle size, 5 *μ*m). The mobile phase consisted of eluent A (0.1% formic acid in water with 10% acetonitrile) and eluent B (acetonitrile). The starting eluent was 100% eluent A. The proportion of eluent B was increased linearly to 36% from 0 min to 60 min, to 60% from 60 min to 90 min, and to 100% from 90 min to 110 min. The detector wavelength was set over a range of 190 to 700 nm and was recorded at 254 nm. The flow rate was 1.0 mL/min, and the injection volume was 20 *μ*L. Identification was based on the retention time and comparison of the obtained spectra with UV spectra from commercial standards. For each compound, the peak area was determined at the wavelength providing maximal UV absorbance.

### 2.4. Cell Viability Assay

BV2 microglia cells were obtained from Professor Hyun Park at Wonkwang University (Iksan, Korea). The cells were maintained at 5 × 10^5^ cells/mL in DMEM medium supplemented with 10% heat-inactivated FBS, penicillin G (100 U/mL), streptomycin (100 mg/mL), and l-glutamine (2 mM) and were incubated at 37°C in a humidified atmosphere containing 5% CO_2_ and 95% air. To determine the cell viability, 50 mg/mL of 3-[4,5-dimethylthiazol-2-yl]-2,5-diphenyltetrazolium bromide (MTT) was added to 1 mL of cell suspension (1 × 10^5^ cells in a 96-well plates) and incubated for 4 h. The obtained formazan was then dissolved in acidic 2-propanol, and optical density was measured at 590 nm.

### 2.5. Nitrite Assay

NO production was assessed using a protocol published by Lee et al. [32]. To determine the NO levels, the concentration of nitrite in the medium was measured using the Griess reaction. The supernatant (100 *µ*L) was mixed with an equal volume of Griess reagent, and the absorbance of the mixture was determined at 525 nm with an ELISA plate reader from BIO-RAD (Hercules, CA, USA).

### 2.6. PGE_2_, TNF-*α*, and IL-1*β* Assay

BV2 microglia were cultured in 24-well plates, preincubated for 12 h with various concentrations of KCHO-1, and then treated for 18 h with LPS. Culture medium was collected, and the concentration of PGE_2_, TNF-*α*, and IL-1*β* was determined using ELISA kits from R&D Systems (Minneapolis, MN, USA) following the manufacturer's instructions.

### 2.7. Western Blotting

BV2 cells were harvested and pelleted at 200 ×g for 3 min, followed by washing with phosphate-buffered saline (PBS). Cells were lysed with 20 mM Tris-HCl buffer (pH 7.4) containing protease inhibitors (0.1 mM phenylmethanesulfonyl fluoride, 5 mg/mL aprotinin, 5 mg/mL pepstatin A, and 1 mg/mL chymostatin). Protein concentration was determined using the Lowry protein assay kit (P5626; Sigma). An equal amount of protein from each sample was resolved using 12% sodium dodecyl sulfate-polyacrylamide gel electrophoresis (SDS-PAGE) and then electrophoretically transferred onto a Hybond enhanced chemiluminescence (ECL) nitrocellulose membrane from Bio-Rad (Hercules, CA, USA). The membrane was blocked with 5% skimmed milk and incubated with anti-HO-1 (1 : 1000 dilution), anti-iNOS (1 : 500 dilution), anti-COX-2 (1 : 1000 dilution), or anti-*β*-actin (1 : 1000 dilution) primary antibodies at 4°C overnight. The immunoreactive bands were visualized using a horseradish peroxidase-conjugated secondary antibody (1 : 1000 dilution) followed by ECL detection from Amersham Pharmacia Biotech (Piscataway, NJ, USA) and were quantitated using Image Gauge* v*3.12 software from Fujifilm (Tokyo, Japan).

### 2.8. Preparation of Nuclear and Cytosolic Fractions

Cells were homogenized (1 : 20, w : v) in PER-Mammalian Protein Extraction Buffer from Pierce Biotechnology (Rockford, IL, USA) containing a protease inhibitor cocktail from EMD Biosciences (San Diego, CA, USA) and 1 mM phenylmethylsulfonyl fluoride. The cytosolic cell fraction was prepared by centrifugation at 15,000 ×g for 10 min at 4°C. Nuclear and cytoplasmic extracts from BV2 cells were prepared using NE-PER nuclear and cytoplasmic extraction reagents. After treatment, cells (3 × 10^6^ cells in a 60 mm dish) were collected and washed with PBS. After centrifugation, cell lysis was performed at 4°C by vigorous shaking for 15 min in RIPA buffer (150 mM NaCl, 1% NP-40, 0.5% sodium deoxycholate, 0.1% SDS, 50 mM Tris-HCl, pH 7.4, 50 mM glycerophosphate, 20 mM NaF, 20 mM ethylene glycol tetra-acetic acid (EGTA), 1 mM dithiothreitol (DTT), 1 mM Na_3_VO_4_, and protease inhibitors). After centrifugation at 14,800 ×g for 15 min, the supernatant was separated and stored at −70°C until further use. Protein content was determined using the bicinchoninic acid (BCA) protein assay kit.

### 2.9. NF-*κ*B DNA Binding

Cells were pretreated for 12 h with the indicated concentrations of KCHO-1 and stimulated for 1 h with LPS (1 *μ*g/mL). The DNA binding activity of NF-*κ*B in the nuclear extracts was measured using the Trans AM kit from Active Motif (Carlsbad, CA, USA), according to the manufacturer's instructions. Briefly, we added complete binding buffer (30 *μ*L; DTT, herring sperm DNA, and Binding Buffer AM3) to each well. We then added 20 *μ*g of nuclear extracts (diluted to 20 *μ*L in complete lysis buffer) from BV2 cells treated with KCHO-1 and stimulated for 1 h with LPS. The plates were incubated for 1 h at room temperature with mild agitation (100 rpm on a rocking platform). After washing each well with wash buffer, 100 *μ*L of diluted NF-*κ*B antibody (1 : 1000 dilution in 1x antibody binding buffer) was added to each well, and the plates were incubated for 1 h with mild agitation. Wells were washed again with wash buffer, and 100 *μ*L of diluted horseradish peroxidase- (HRP-) conjugated antibody (1 : 1000 dilution in 1x antibody binding buffer) was added to each of the 6 wells. The plates were then incubated for 1 h with mild agitation. Developing solution was added to each well for 5 min, followed by a wash to remove the supernatant. The absorbance was read on a spectrophotometer at 450 nm within 5 min.

### 2.10. RT-PCR Analysis

Total RNA was isolated from cells using Trizol from Invitrogen (Carlsbad, CA, USA) following the manufacturer's instructions and quantified spectrophotometrically at 260 nm. Total RNA (1 *μ*g) was reverse-transcribed using the High Capacity RNA-to-cDNA kit from Applied Biosystems (Carlsbad, CA, USA). The cDNA was then amplified using the SYBR Premix Ex Taq kit from TaKaRa Bio Inc. (Shiga, Japan) and a StepOnePlus Real-Time PCR system from Applied Biosystems. RT-PCR was performed in a total volume of 20 *μ*L, consisting of 10 *μ*L SYBR Green PCR Master Mix, 0.8 *μ*M of each primer, and diethyl pyrocarbonate- (DEPC-) treated water. The primer sequences were designed using PrimerQuest from Integrated DNA Technologies (Cambridge, MA, USA). The primer sequences were as follows:* mHO-1*, forward 5′-CTCTTGGCTGGCTTCCTT-3′, reverse 5′-GGCTCCTTCCTCC TTTCC-3′, and* mGAPDH*, forward 5′-ACTTTGGTATCGTGGAAGGACT-3′, reverse 5′-GTAGAGGCAGGGATGATGTTCT-3′. The optimal conditions for PCR amplification of cDNA were established using the manufacturer's instructions. The mRNA data were analyzed using PCR device of Applied Biosystems (Carlsbad, CA, USA). In addition, the data were analyzed also using StepOne software from Applied Biosystems (Carlsbad, CA, USA), and the cycle number at the linear amplification threshold (Ct) values for the endogenous control* mGAPDH* and the target gene were recorded. Relative gene expression (target gene expression normalized to the expression of the endogenous control gene) was calculated using the comparative Ct method (2^−ΔΔCt^).

### 2.11. Statistical Analysis

Data were expressed as the mean ± SD of at least 3 independent experiments. To compare 3 or more groups, one-way analysis of variance (ANOVA) was used followed by Newman-Keuls* post hoc* test. Statistical analysis was performed using GraphPad Prism software version 3.03 from GraphPad Software Inc. (San Diego, CA, USA).

## 3. Results

### 3.1. HPLC Analysis of KCHO-1 and the Effects of KCHO-1 on Proinflammatory Enzyme Expression and Mediator Production in LPS-Stimulated BV2 Microglia

**Figure 1:**
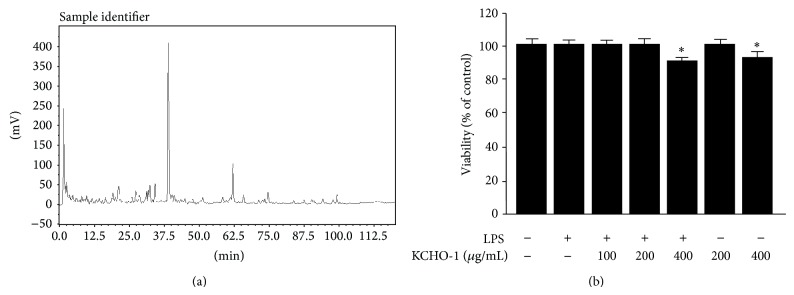
HPLC chromatograms of KCHO-1 (a) and the effects on cell viability (b) of BV2 microglia. Chromatograms were obtained via HPLC using KCHO-1 dissolved in acetonitrile at a concentration of 10 mg/mL. The method utilized a hydro column at a flow rate of 1 mL/min with a gradient of 0-36-60-100% acetonitrile for 110 min and an HPLC-DAD (Sykam) that read at 254 nm. The UV spectra peak at 38.858 min was identified as the major component of KCHO-1 (a). BV2 microglia were incubated for 48 h with the indicated concentrations of KCHO-1, or pretreated with the indicated concentrations of compounds KCHO-1 for 12 h, and then stimulated with LPS (1 *μ*g/mL) for 18 h (b). The data represent the mean values from 3 experiments ± SD. ^*^ *P* < 0.05 compared with the nontreated control group.

**Figure 2:**
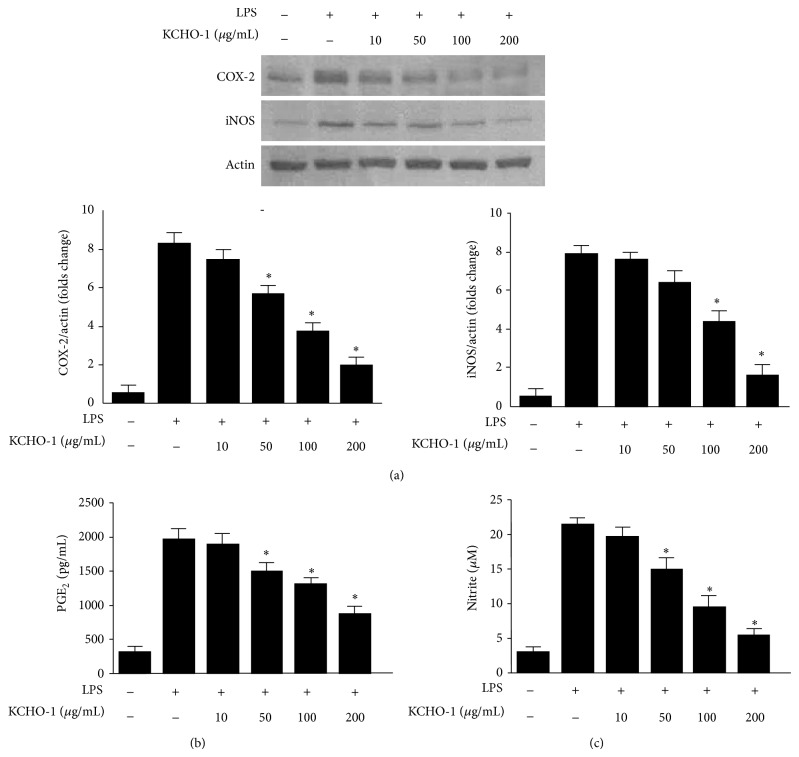
The effects of KCHO-1 on COX-2 and iNOS (a) expression and PGE_2_ (b) and NO (c) production in LPS-stimulated BV2 microglia. The cells were pretreated for 12 h with the indicated concentrations of KCHO-1 and stimulated for 18 h with LPS (1 *μ*g/mL). Western blot analyses (a) were performed as described in the Materials and Methods, and representative blots from 3 independent experiments are shown. The concentration of PGE_2_ (b) and NO (c) was determined as described. The data represent the mean values from 3 experiments ± SD. ^*^ *P* < 0.05 compared with the LPS-treated group.

Data from the HPLC analysis of KCHO-1 was obtained in the form of chromatograms by monitoring detector responses at 254 nm. As shown in Figure 1(a), the retention time of the main peak was 38.858 min. Next, we evaluated the cytotoxicity of KCHO-1 on BV2 microglia by using an MTT assay. As shown in Figure 1(b), cell viability was not significantly altered up to 400 *µ*g/mL of KCHO-1. Therefore, for all subsequent experiments, the concentration ranges of KCHO-1 were maintained between 10 and 200 *µ*g/mL. To investigate the effects of KCHO-1 on iNOS and COX-2 expression, and on proinflammatory mediators, BV2 microglia cells were treated with varying concentrations of KCHO-1 (10–200 *μ*g/mL) for 12 h, followed by LPS stimulation. Pretreatment of the BV2 microglia with KCHO-1 for 12 h resulted in decreased COX-2 and iNOS expression (Figure 2(a)). Under the same conditions, we found that KCHO-1 reduced COX-derived PGE_2_ and iNOS-derived NO production in a concentration-dependent manner (Figures 2(b) and 2(c)).

### 3.2. Effects of KCHO-1 on Proinflammatory Cytokine Production in LPS-Stimulated BV2 Microglia

**Figure 3:**
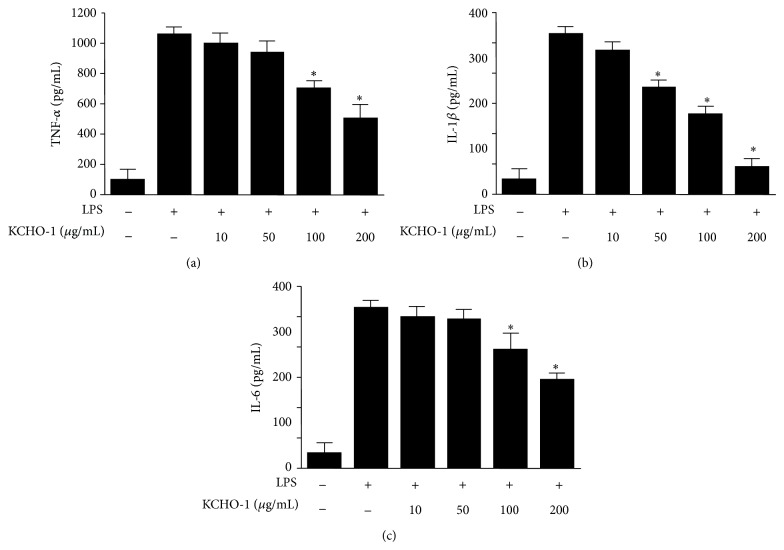
The effects of KCHO-1 on TNF-*α* (a), IL-1*β* (b), and IL-6 (c) production in LPS-stimulated BV2 microglia. The cells were pretreated for 12 h with the indicated concentrations of KCHO-1 and then stimulated for 18 h with LPS (1 *μ*g/mL). The concentrations of TNF-*α* (a), IL-1*β* (b), and IL-6 (c) were determined using ELISA kits, as described in the Materials and Methods section. The data represent the mean values from 3 experiments ± SD. ^*^ *P* < 0.05 compared with the LPS-treated group.

Our results demonstrated that KCHO-1 suppressed LPS-stimulated proinflammatory mediators such as NO, PGE_2_, iNOS, and COX-2. We further investigated the effects of KCHO-1 on LPS-induced TNF-*α*, IL-1*β*, and IL-6 production. The cells were pretreated with KCHO-1 for 12 h and then stimulated with LPS. Enzyme immunoassay data indicated that KCHO-1 reduced TNF-*α*, IL-1*β*, and IL-6 production in a dose-dependent manner (Figure 3).

### 3.3. Effects of KCHO-1 on the Degradation of I*κ*B-*α*, NF-*κ*B Nuclear Translocation, and NF-*κ*B DNA Binding in LPS-Stimulated BV2 Microglia

**Figure 4:**
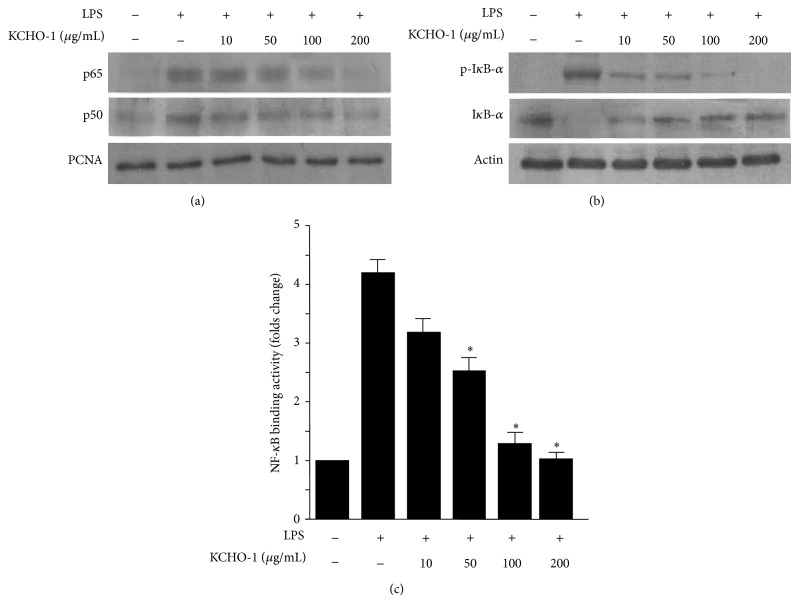
The effects of KCHO-1 on NF-*κ*B activation (a), I*κ*B-*α* phosphorylation, degradation of I*κ*B-*α* (b), and NF-*κ*B DNA binding activity (c) in BV2 microglia. The cells were pretreated for 12 h with the indicated concentrations of KCHO-1 and stimulated for 1 h with LPS (1 *μ*g/mL). Western blot analysis of I*κ*B-*α* and p-I*κ*B-*α* in the cytoplasm and NF-*κ*B in the nucleus (a, b) was performed as described in the Materials and Methods. A commercially available NF-*κ*B enzyme-linked immunosorbent assay (ELISA) kit (Active Motif) was used to test nuclear extracts and determine the degree of NF-*κ*B binding (c). The data represent the mean values from 3 experiments ± SD. ^*^ *P* < 0.05 compared with the LPS-treated group.

Previous studies have reported that NF-*κ*B is a crucial transcription factor that regulates the production of proinflammatory mediators. We therefore investigated the effects of KCHO-1 on the NF-*κ*B signaling pathway. As shown in Figure 4(a), KCHO-1 significantly inhibited the translocation of p65 and p50, the two subunits of the NF-*κ*B heterodimer, to the nucleus. In addition, KCHO-1 inhibited the phosphorylation and degradation of I*κ*B-*α* in the cytoplasm (Figure 4(b)). Furthermore, as shown in Figure 4(c), we examined the ability of NF-*κ*B to bind DNA in the nuclear extracts from BV2 microglia induced with LPS for 1 h. LPS treatment markedly increased the amount of NF-*κ*B bound to DNA; however, KCHO-1 inhibited NF-*κ*B binding to DNA in a dose-dependent manner.

### 3.4. Effects of KCHO-1 on HO-1 mRNA and Protein Expression and Nuclear Translocation of Nrf2 in BV2 Microglia

**Figure 5:**
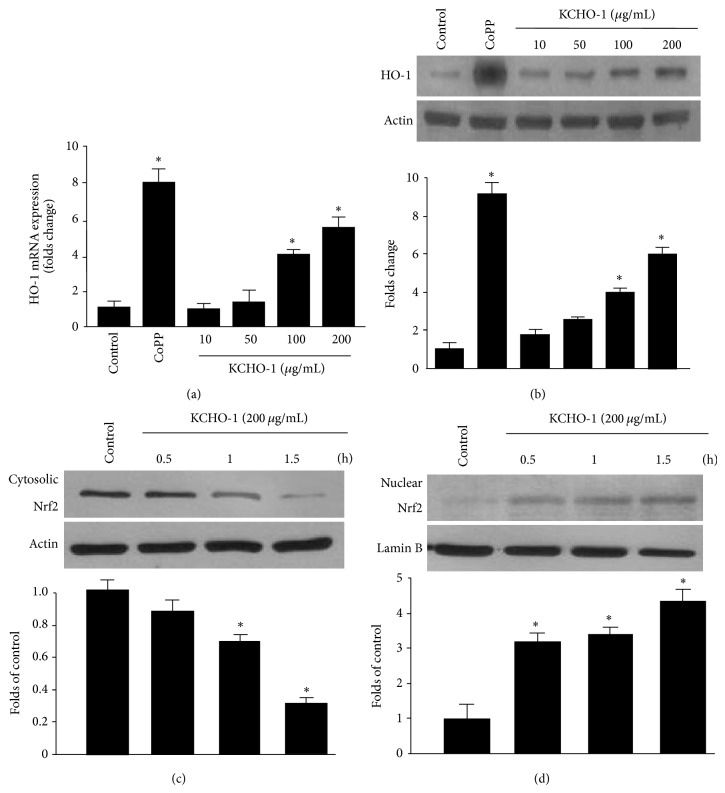
The effects of KCHO-1 on HO-1 mRNA (a) and protein (b) expression and the nuclear translocation of Nrf2 (c, d) in BV2 microglia. Cells were incubated for 12 h with the indicated concentrations of KCHO-1 (a, b) or treated with 200 *μ*g/mL of KCHO-1 for 0.5, 1, and 1.5 h (c, d). RNA quantification and western blot analysis for* mHO-1* and* mGAPDH* expression were performed, and the nuclei were fractionated from the cytosol using PER-Mammalian Protein Extraction Buffer, as described in the Materials and Methods. The blots are representative of 3 independent experiments. ^*^ *P* < 0.05 compared with the control group.

We next examined the effects of KCHO-1 on HO-1 expression in BV2 microglia. The cells were treated with noncytotoxic concentrations of KCHO-1 (10–200 *μ*g/mL) for 12 h. Our data showed that KCHO-1 induced a dose-dependent increase in the* mHO-1* mRNA expression (Figure 5(a)) and HO-1 protein expression (Figure 5(b)). KCHO-1 and the positive control CoPP, which induces HO-1 expression, dose-dependently increased* mHO-1* (Figure 5(a)) and HO-1 protein expression (Figure 5(b)). Nuclear translocation of activated Nrf2 regulates HO-1 mRNA and protein expression. Therefore, we investigated whether KCHO-1 treatment induced the nuclear translocation of Nrf2 in BV2 microglia (Figures 5(c) and 5(d)). Our results demonstrated that the nuclear fraction of BV2 microglia showed a gradual increase in Nrf2 levels, with a simultaneous decrease in cytoplasmic Nrf2 levels.

### 3.5. HO-1 Mediates the Effects of KCHO-1 on Proinflammatory Mediators and Cytokines and NF-*κ*B Binding Activity in LPS-Stimulated BV2 Microglia

**Figure 6:**
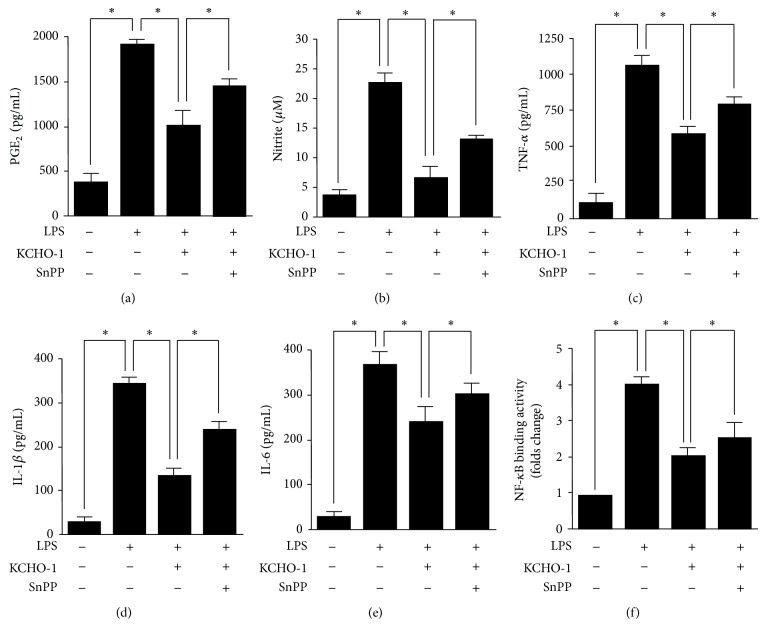
HO-1 mediates the suppressive effect of KCHO-1 on LPS-stimulated proinflammatory mediator production and NF-*κ*B DNA binding. BV2 microglia were pretreated for 12 h with KCHO-1 (200 *μ*g/mL), in the presence or absence of SnPP (50 *μ*M), and stimulated for 18 h (a, b, c, d, e) or 1 h (f) with LPS (1 *μ*g/mL). The concentrations of PGE_2_ (a), NO (b), TNF-*α* (c), IL-1*β* (d), and IL-6 (e) were determined using ELISA kits, as described in the Materials and Methods. A commercially available NF-*κ*B enzyme-linked immunosorbent assay (ELISA) kit (Active Motif) was used to test nuclear extracts and determine the degree of NF-*κ*B binding (f). Cells were pretreated with SnPP for 3 h in this experiment. The data represent the mean values from 3 experiments ± SD. ^*^ *P* < 0.05.

To confirm that the observed antineuroinflammatory effects were mediated by KCHO-1-induced expression of HO-1, we investigated the effect of SnPP, a competitive HO inhibitor, on proinflammatory mediators and cytokine levels. Cells were pretreated with KCHO-1 (200 *μ*g/mL) for 12 h in the presence or absence of SnPP (50 *μ*M). Our results showed that the inhibitory effects of KCHO-1 on LPS-induced PGE_2_, NO, TNF-*α*, IL-1*β*, and IL-6 production were partially reversed by SnPP. Furthermore, we investigated whether the upregulation of HO-1 by KCHO-1 mediated NF-*κ*B inhibition (Figure 6). Cells were pretreated with KCHO-1 for 12 h (20 *μ*g/mL) in the presence or absence of SnPP. Then, the cells were stimulated for 1 h with LPS (1 *μ*g/mL), and the nuclear extracts were obtained. NF-*κ*B binding was observed in the nuclear extracts from BV2 microglia. However, SnPP markedly reduced the inhibitory effect of KCHO-1 on NF-*κ*B DNA binding.

## 4. Discussion

Neurodegeneration refers to the progressive loss of neuronal structure and function, leading to neuronal death. Many neurodegenerative diseases, including PD, AD, and Huntington's diseases, occur because of neurodegenerative processes. Brain tissues are vulnerable to oxidative stress and inflammation that may occur through physiological or pathological processes [33]. Our lab has focused on the identification of natural products that modulate HO-1 activity and investigating the molecular mechanisms of these effects in neurodegenerative diseases [32, 34]. In the present study, we investigated the potential involvement of HO-1 expression, regulated by nuclear translocation of Nrf2, in the antineuroinflammatory activity of KCHO-1. KCHO-1 is a novel herbal combination composed of 30% EtOH extracts obtained from nine herbal medicines, including* Curcuma longa*,* Salvia miltiorrhiza*,* Gastrodia elata*,* Chaenomeles sinensis*,* Polygala tenuifolia*,* Paeonia japonica*,* Glycyrrhiza uralensis*,* Atractylodes japonica*, and processed* Aconitum carmichaeli*. It was well known that* C. longa* has antitumor, anti-inflammatory, antioxidative, and hepatoprotective effects [16, 17].* S. miltiorrhiza* exhibits algicidal activity and anti-inflammatory activity [18, 19].* G. elata* possesses neuroprotective and antineuroinflammatory effects [20, 21].* C. sinensis* and* P. tenuifolia* have antioxidant, antidiabetic, and anti-inflammatory effects [22–25].* P. japonica* has been shown to prevent fatigue [26], and* G. uralensis* has antiasthma and antibacterial activity [27, 28].* A. japonica* exhibits antiallergic and anti-inflammatory effects [29, 30], and processed* A. carmichaeli* has pain-relieving effects [31]. These herbs have been used in traditional medicine for many years. In this study, we have investigated the antineuroinflammatory effects of KCHO-1 in LPS-induced BV2 microglia to determine its therapeutic potential in neuroinflammation.

Neuroinflammation is a characteristic pathologically observed in several neurodegenerative disorders [35]. Microglia are the resident macrophages of the brain and are activated upon brain injury, thereby releasing various proinflammatory cytokines and inflammatory mediators in the CNS [36]. The BV2 immortalized murine microglia cell line is widely used as a model of microglia* in vitro* because BV2 cells retain most of the morphological and functional properties described for primary microglia. To investigate the effects of KCHO-1 on the production of proinflammatory mediators and the expression of iNOS and COX-2, BV2 microglia were stimulated with LPS in the presence or absence of noncytotoxic concentrations of KCHO-1. We showed that KCHO-1 inhibited iNOS and COX-2 protein expression, thereby suppressing COX-2-derived PGE_2_, iNOS-derived NO, TNF-*α*, IL-1*β*, and IL-6 productions. NF-*κ*B signaling is regarded as a key mediator of the immune response in the brain, including neuronal and glial cells [37]. Upon activation, the p50 and p65 subunits of the free NF-*κ*B dimer translocate into the nucleus and bind to specific DNA sequences in the promoter regions of proinflammatory enzymes and cytokines [38]. To investigate whether NF-*κ*B activation and DNA binding activity could be an upstream target for the inhibitory effects of KCHO-1, we examined the effects of KCHO-1 on the phosphorylation of I*κ*B-*α* and the nuclear translocation of the NF-*κ*B subunits p65 and p50. Our results clearly showed that LPS-induced phosphorylation and degradation of I*κ*B-*α* and nuclear translocation of p65 and p50 were significantly reduced after KCHO-1 pretreatment of BV2 microglia. KCHO-1 also dose-dependently inhibited the increase in NF-*κ*B DNA binding in LPS-stimulated BV2 microglia. These findings suggest that KCHO-1, at least in LPS-stimulated microglia, exerted its antineuroinflammatory effects by inhibiting NF-*κ*B activation, thereby reducing the expression of the proinflammatory enzymes iNOS and COX-2 and suppressing the secretion of the proinflammatory cytokines NO, PGE_2_, TNF-*α*, IL-1*β*, and IL-6.

The Nrf2/ARE pathway and phase 2 antioxidant enzymes, including HO-1, have emerged as a therapeutic target for neuronal protection [39, 40]. Along with its antioxidative effects, recent studies have shown that HO-1 has anti-inflammatory effects in microglia using a number of inflammatory models [41]. HO-1 and its product, carbon monoxide, can also suppress the expression of proinflammatory mediators, cytokines, and chemokines [10–13]. Thus, the identification of constituents in natural products that have neuroprotective and antineuroinflammatory effects through Nrf2/ARE-mediated HO-1 expression would be valuable for therapeutic application in neurodegenerative diseases. The results of this study provide evidence that HO-1 mRNA and protein expression is dose-dependently induced by KCHO-1 in BV2 microglia. KCHO-1 also induced Nrf2 levels in the nucleus of BV2 microglia. In addition, our results indicate that the inhibition of HO activity by the HO inhibitor SnPP reversed the inhibitory effects of KCHO-1 in LPS-stimulated microglia. Because SnPP blocks HO enzymatic activity, these data confirmed that KCHO-1 could inhibit NO, PGE_2_, TNF-*α*, IL-1*β*, and IL-6 production and NF-*κ*B DNA binding through modulation of HO-1 expression. Therefore, these data indicate that KCHO-1-induced HO-1 expression was at least partially responsible for the resulting antineuroinflammatory effects.

In conclusion, we demonstrated that KCHO-1, a novel combination of 30% EtOH herbal extracts, increased HO-1 mRNA and protein expression via the Nrf2 pathway. In addition, KCHO-1-induced HO-1 expression suppressed NF-*κ*B-mediated production of proinflammatory mediators, cytokines, and protein in LPS-stimulated microglia. Thus, KCHO-1 may represent a potential natural therapeutic for the treatment of neuroinflammation.
